# Adiponectin Impairs Chicken Preadipocytes Differentiation through p38 MAPK/ATF-2 and TOR/p70 S6 Kinase Pathways

**DOI:** 10.1371/journal.pone.0077716

**Published:** 2013-10-23

**Authors:** Jun Yan, Lu Gan, Di Chen, Chao Sun

**Affiliations:** College of Animal Science and Technology, Northwest A&F University, Yangling, Shaanxi, China; Virgen Macarena University Hospital, School of Medicine, University of Seville, Spain

## Abstract

Adiponectin is a protein hormone secreted exclusively by adipocytes that plays an important role in the modulation of glucose and lipid metabolism. In the present study, we investigated the ability of adiponectin to stimulate chicken preadipocyte differentiation and its effect on cellular signaling pathways associated with adipocyte differentiation. Data showed that over-expression of adiponectin inhibited adipocyte differentiation and the expression of adipogenic marker gene, while activated the expression of lipolytic marker gene. Meanwhile, adiponectin led to activation of p38 mitogen-activated protein kinase (p38 MAPK)/activating transcription factor 2 (ATF-2) signaling pathway and down-regulation of target of rapamycin (TOR)/p70 S6 Kinase signaling pathway. Furthermore, the activation of p38 MAPK/ATF-2 signaling pathway was blocked by the p38 MAPK inhibitor SB253580, whereas adiponectin had a synergistic effect on the suppression of TOR/p70 S6 Kinase signaling pathway with the TOR inhibitor rapamycin. In conclusion, the results demonstrate the ability of adiponectin to inhibit chicken preadipocyte differentiation, which depends on the p38 MAPK/ATF-2 and TOR/p70 S6 Kinase pathways.

## Introduction

Adiponectin (ADPN) is a 30 kDa adipokine hormone secreted from adipose tissue, which consists of an N-terminal collagenous domain and a C-terminal globular domain as the receptor binding effector [1], [2]. It plays a fundamental role in lipid and carbohydrate metabolism. ADPN stimulates fatty acid oxidation, decreases plasma triglycerides, improves glucose metabolism and increases insulin sensitivity [3]. Studies also indicated that circulating adiponectin levels were reduced in patients with insulin resistance, type2 diabetes, obesity, or cardiovascular disease [4], [5], [6]. Recently, researchers also found that the level of adiponectin was negatively related to chicken belly fat deposition level [7]. However, the function of adiponectin on the differentiation of adipocytes remains controversial. Fu et al (2005) reported that adiponectin could promote adipocyte differentiation, insulin sensitivity, and lipid accumulation [8]. In contrast, Bauche et al (2007) proved that mice over-expressing adiponectin specifically in white fat showed a clear reduction in adiposity due to increased energy expenditure and to impaired adipocyte differentiation [9]. Thus, further research is required to elucidate adiponectin's metabolic effects and mechanism of the action.

p38 mitogen-activated protein kinase (MAPK) is a major kinase in the MAPK family and plays an essential role in regulating cell proliferation, inflammation and immune responses [10]. Recent studies suggested that p38 MAPK acted as an essential mediator in regulating adiponectin-induced glucose uptake and fatty acid oxidation in mouse C2C12 myotubes and also played a negative role in adipogenesis via the inhibition of CCAAT/enhancer binding protein beta (C/EBPβ) and peroxisome proliferator-activated receptor gamma (PPARγ) transcriptional activities [11], [12], [13]. Activating transcription factor 2 (ATF-2), a ubiquitously expressed member of the cyclic adenosine monophosphate (cAMP) -responsive element binding protein family of basic region leucine-zipper transcription factors, has been implicated in multiple responses in mammalian cells by regulating the expression of a broad spectrum of target genes [14]. Maekawa et al (2007) indicated that ATF-2 family transcription factors played a role in adipocyte differentiation and fat storage [15]. Target of rapamycin (TOR), an evolutionarily conserved serine/threonine protein kinase, is a major effector of adipocyte differentiation via the regulation of adipogenesis [16]. Xin X et al (2011) demonstrated that the TAK1-MKK3 cascade mediated adiponectin signaling and uncovered a scaffolding role of APPL1 in regulating the TAK1-MKK3-p38 MAPK pathway in C2C12 cells [11]. Sugiyama et al (2009) indicated that adiponectin inhibited colorectal cancer cell growth via activation of AMP-activated protein kinase (AMPK), thereby down-regulating the mTOR pathway. However, it is still not clear that if the p38 MAPK and TOR signaling pathways are involved in the process of chicken preadipocytes differentiation induced by adiponectin.

In this study, chicken *adiponectin* gene over-expression and interference vectors were constructed and transfected into chicken preadipocytes. The ability of adiponectin to stimulate adipocyte differentiation and the related signaling pathways were investigated. Our results demonstrated that adiponectin inhibited chicken preadipocyte differentiation via the activation of p38 MAPK/ATF-2 and the suppression of TOR/p70 S6 Kinase signaling pathways.

## Materials and Methods

### Chicken preadipocytes culture

Twenty male Cobb broilers were used as a source of adipose tissue. Chicks were maintained on a 24 h constant-light schedule and allowed *ad libitum* access to experimental diets (22% crud protein) and water. Abdominal and groin adipose tissues were collected from 7 to 12-day-old chicks by sterile dissection after anesthetized with intraperitoneal injection of 50 mg/kg barbiturate, and the samples from different chicks in each experiment were pooled together. All experimental procedures were approved by the Animal Ethics Committee of Northwest A&F University. Visible fibers and blood vessels were removed and the adipose tissue was washed three times with PBS buffer containing 200 U/ml penicillin (Sigma, USA) and 200 U/ml streptomycin (Sigma, USA). Then the adipose tissue was minced into fine sections (1 mm^3^) with scissors and incubated in 10 ml of digestion buffer containing DMEM/F-12 (Gibco, USA), 100 mM HEPES (Sigma, USA), 1.5% bovine serum albumin (Sigma, USA), 2 mg/ml type I collagenase (Sigma, USA) for 50 min at 37 °C in a water bath. After the incubation, growth medium (DMEM/F-12 (50∶50), 10% fetal bovine serum (Sigma, USA), 100 U/ml penicillin and 100 U/ml streptomycin) was added to the digestion flask. Flask contents were mixed and filtered through nylon screens with 250 and 20 µm mesh openings to remove undigested tissue and large cell aggregates. The filtered cells were centrifuged at 1,300 rpm for 7 min to separate floating adipocytes from cell pellets. Cell pellets were incubated in 5 ml erythrocyte splitting liquor (0.154 M NH_4_Cl, 10 mM KHCO_3_, 0.1 mM EDTA) for 10 min, and then centrifuged at 1,300 rpm for 7 min. Cells were then seeded in 60-mm culture dish at a density of 1×10^4^ cells/cm^2^ and cultured in a humidified atmosphere of 95% air and 5% CO_2_ at 37 °C until confluence. The medium was changed every other day.

### Plasmid construction and transient transfection

Total RNA was extracted from chicken abdomen fat. After *adiponectin* (GeneBank, NM_206991) gene was cloned, eukaryotic expression vector pcDNA 3.1-ADPN was constructed. Small-interfering RNA (siRNA) against *ADP*N was contrived based on the principle of siRNA construction and synthesized by Invitrigen Company (Shanghai, China) using pGPU6/GFP/Neo siRNA expression vector (Genepharma USA). Three sequences were designed as the target interfering segments and described as follows: siRNA-1: pGPU6/GFP/Neo-ADPN-676 (bases 676–696): (forward 5′- CAC CGC TTC ACC AAG ATC TTC TAC ATT CAA GAG ATG TAG AAG ATC TTG GTG AAG CTT TTT TG-3′, reverse 5′- GAT CCA AAA AAG CTT CAC CAA GAT CTT CTA CAT CTC TTG AAT GTA GAA GAT CTT GGT GAA GC-3′); siRNA-2: pGPU6/GFP/Neo-ADPN-751 (bases 751-771): (forward 5′- CAC CGC ACG TAC TTC TTT GCC TAC CTT CAA GAG AGG TAG GCA AAG AAG TAC GTG CTT TTT TG-3′, reverse 5′- GAT CCA AAA AAG CAC GTA CTT CTT TGC CTA CCT CTC TTG AAG GTA GGC AAA GAA GTA CGT GC-3′); siRNA-3: pGPU6/GFP/Neo-ADPN-952 (bases 952–972): (forward 5′- CAC CGG GTC TAT GCT GAC AAC ATC ATT CAA GAG ATG ATG TTG TCA GCA TAG ACC CTT TTT TG -3′, reverse 5′- GAT CCA AAA AAG GGT CTA TGC TGA CAA CAT CAT CTC TTG AAT GAT GTT GTC AGC ATA GAC CC-3′). Glyceraldehyde 3-phosphate dehydrogenase (GAPDH) was used as positive control and pGPU6/GFP/Neo-siRNA-GAPDH was also constructed (siGH): (forward 5′- CAC CGT ATG ACA ACA GCC TCA AGT TCA AGA GA C TTG AGG CTG TTG TCA TAC TTT TTT G-3′, reverse 5′-GAT CCA AAA AA G TAT GAC AAC AGC CTC AAG TCT CTT GAA CTT GAG GCT GTT GTC ATA C-3′). Sub-cultured chicken adipocytes were cultured in 35-mm culture dishes, the cells were subjected to transient transfection using Lipofectamine reagent (Invitrogen, CA) according to the manufacturer's instructions when the density reached 70 to 80 percent. A 2:1 ratio of lipofectamine-vector complexes were prepared and added.

### Experimental design

Eukaryotic expression vector pcDNA 3.1-ADPN (pA) and interference vectors (siRNA-1, siRNA-2 and siRNA-3) were used to study adiponectin gene's function. The transcriptional level of *ADP*N gene was detected at 24 h after transfection with plasmids and ADPN protein was detected at day 2, 3 and 9. After the analysis of interference efficiency, we chose one interference vector with highest interference efficiency for further research. 10 µM SB203580 (Sigma, USA) and 10 nM rapamycin (Sigma, USA) were used to treat cells at 36 h after transfection with plasmids. 30 min after administration of drugs, cellular proteins were collected to check the phosphorylation levels of p38 MAPK or TOR pathway marker proteins. Oil Red O staining and the detection of lipid metabolism in adipocytes were taken at day 1, 3 and 9 after transfection; the transcriptional levels of metabolism-related genes were examined at day 1, 3 and 9; lipid metabolism-related proteins were evaluated at day 3 and 9. Each test was repeated six times with mixed cell cultures derived from different chicks and this experiment was repeated three separate times.

### Oil Red O staining of adipocytes and analysis of lipid metabolism

Cytoplasmic lipid droplets were detected by Oil Red O staining (Invitrogen, CA) 1, 3 and 9 day after transfection. Briefly, the cells were washed three times in PBS buffer and then fixed in 10% (v/v) formaldehyde for 30 min. The fixed cells were then washed three times in PBS buffer and stained with a working solution of Oil Red O for 30 min at room temperature. The cells were then washed with deionized water, and extracted by 100% avantin for colorimetric analysis at 510 nm using an inverted microscope (Nikon Instruments Europe BV, England). Triglyceride (TG) accumulation was displayed with absorbance value at 510 nm. The area of the cells stained with Oil Red O and the droplet diameter frequency distributions were measured by Image-Pro Plus analyzer (Palmerton, USA). The medium was removed from each well at day 1, 3, 9 and assayed for glycerol content at absorption at 540 nm by use of a free glycerol determination kit (Sigma, USA) and free fatty acid (FFA) content was determined at absorption at 570 nm with a FFA assay kit (Jiancheng, China).

### Real-time quantitative PCR analysis

RNA was extracted with TRIpure Reagent kit (Takara, Japan) and 400 ng of total RNA was reverse transcribed using the M-MLV reverse transcriptase kit (Takara, Japan). Primers for *PPARγ*, C/EBPα, *fatty acid synthase (FAS)*, *adipose triglyceride lipase (ATGL)* and adiponectin were synthesized by Shanghai Sangon Biological Engineering Technology and Service Co., Ltd. Primer sequences for genes are shown in Table 1. *Glyceraldehyde 3-phosphate de-hydrogenase (GAPDH)* and *β-actin* were used as internal control in PCR amplification. Quantitative PCR was performed in 25 µl reactions containing SYBR Premix EX Taq (Takara) 12.5 µl, upstream and downstream primer each 0.5 µl, cDNA 1.0 µl, deionized water 10.5 µl. Reaction mixtures were incubated for initial denaturation at 95 °C for 10 min, followed by 40 cycles, each cycle consisting of 95 °C for 15 s and 60 °C for 1 min. A melting curve analysis was incorporated at the end of each run to control for amplification specificity. The levels of mRNAs were normalized to *β-actin*. The expression of genes were analyzed by method of 2^−△△Ct^ [17].

**Table 1:** List of genes examined and their corresponding accession numbers.

Gene name	Primer sequences (5′-3′)	Product size (bp)	Accession number
*β-actin*	F: GGCTGTGCTGTCCCTGTATGC	207	NM_205518
	R: CTCTCGGCTGTGGTGGTGAAG		
*GAPDH*	F:GGTGGTGCTAAGCGTGTTA	179	NM_204305
	R: CCCTCCACAATGCCAA		
*PPARγ*	F: CGAATGCCACAAGCGGAGAAGG	147	NM_001001460
	R: CTTGGCTTTGGTCAGCGGGAA		
*C/EBPα*	F: ATGGAGCAAGCCAACTTCTAC	230	NM_001031459
	R: GCCAGGAACTCGTCGTTGAA		
*FAS*	F: GGCTGGAAGGAGAGTGATGGG	216	NM_205155
	R: ACACCTCCTGAGCCAGAGTGAATG		
*ATGL*	F: GGACTCCGCTTGGAACATCTC	229	NM_001113291
	R: GAACCTCTTTCGTGCTTCTTTGG		
*Adiponectin*	F: AAGGAGAGCCAGGTCTACAAGGTG	238	NM_206991
	R: GTGCTGCTGTCGTAGTGGTTCTG		

Footnote: F and R indicate forward and reverse primers respectively. *GAPDH  =  Glyceraldehyde 3-phosphate de-hydrogenase*, *C/EBPα  =  CCAAT/enhancer binding protein alpha*, *PPARγ  =  Peroxisome proliferator-activated receptor gamma*, *FAS  =  fatty acid synthase, ATGL  =  adipose triglyceride lipase*.

### Western blot analysis

Whole cell extracts were prepared using 150 µl/35 mm-dish lysis buffer containing 20 mM Tris, pH 7.5, 5 mM EGTA, 150 mM NaCl, 1% Nonidet P-40, 0.1 mM Na_3_VO_4_, 1 mM NaF, 10 Mm sodium β-glycerophosphate, 0.1 mM phenylmethylsulfonyl fluoride,1 µg/ml leupeptin, 10 µg/ml aprotinin for 30 min at 4 °C. The lysate was clarified by centrifugation at 10,000 × g for 10 min at 4 °C, and the supernatants were used to assay protein levels. Total protein extracts (0.05 g) were separated by electrophoresis on 12% and 5% SDS-PAGE gels using slab gel apparatus and then transferred to PVDF nitrocellulose membranes. After transfer, the membranes were blocked in blocking buffer (1% Skim Milk Powder, 10 mM tris, 150 mM NaCl and 0.02% Tween 20) for 2 h at room temperature. Membranes were then incubated with primary antibodies against β-actin, PPARγ, C/EBPα, FAS, ATGL (Santa Cruz, USA) and p38 MAPK, phospho-p38 MAPK (pT180/pY182), ATF-2, phospho-ATF-2 (pT71), TOR, phospho-TOR (pS2448), p70 S6 Kinase, phospho-p70 S6 Kinase (pT421/pS424) (Epitomic, USA) at 4 °C overnight and incubated with the appropriate HRP-conjugated secondary antibodies for 2 h at room temperature. Proteins were visualized using chemiluminescent peroxidase substrate (millipore), and then the blots were quantified using ChemiDoc XRS system (Bio-Rad) and analysis software Quantity One (Bio-Rad).

### Statistics

Statistical calculations were performed with SAS v8.0 (SAS Institute, Cary, NC). Statistical significance was determined using the ANOVA regression table. Comparisons among individual means were made by Fisher's least significant difference (LSD) post hoc test after ANOVA. Data are presented as mean± SEM. *P*<0.05 was considered to be statistically significant.

## Results

### Efficiency detection of the expression of *adiponectin* gene in chicken adipocytes

**Figure 1:**
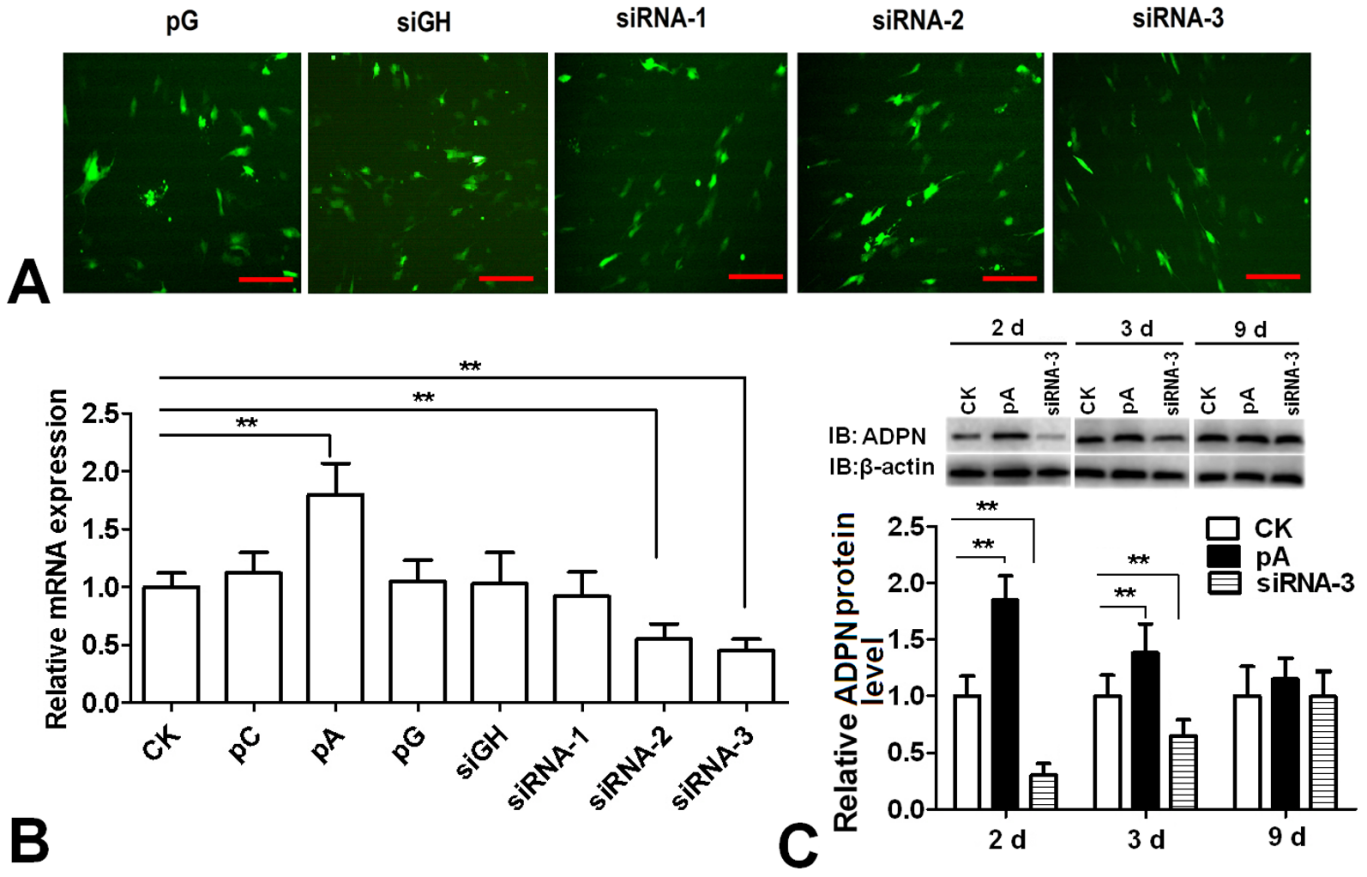
Efficiency detection of adiponectin expression in chicken preadipocytes. (A) GFP observed by fluorescence microscopy 24 h after transfection with pGPU6 recombinant vectors. Scale bar, 100 µm. (B) The expression of *adiponectin* gene 24 h after transfection with pcDNA3.1-ADPN and pGPU6 recombinant vectors (n = 3). (C) The expression of adiponectin protein at day 2, 3 and 9 after transfection with pcDNA3.1-ADPN and siRNA-3 (n = 3). CK: Control group, pC: pcDNA3.1, pA: pcDNA3.1-ADPN, pG: pGPU6/GFP/Neo, siRNA-1: pGPU6/GFP/Neo-ADPN-676, siRNA-2: pGPU6/GFP/Neo-ADPN-751, siRNA-3: pGPU6/GFP/Neo-ADPN-952, siGH: pGPU6/GFP/Neo- siGAPDH. Values are means ± SEM. vs. control group, * *P*<0.05, ** *P*<0.01.

As shown in **Fig. 1A**, 24 h after transfection with pGPU6/GFP/Neo recombination vectors, GFP protein was observed by fluorescence microscopy, which indicated that there was similar transfection efficiency among the groups. Compared to the control group, the expression of *ADP*N gene increased 80% in the pcDNA 3.1-ADPN transfection group, while decreased 57% and 66% in the siRNA-2 and siRNA-3 transfection groups respectively (*P<*0.01). There was no significant change in the siRNA-1 transfection group (**Fig. 1B**). Therefore, siRNA-3 was chosen for the subsequent experiment due to its higher interference efficiency. Western Blot was used to confirm the translation level of ADPN (**Fig. 1C**). Compared to control group, the expression of ADPN protein significantly increased in the pcDNA3.1-ADPN transfection group at day 2 and 3, while decreased in the siRNA-3 transfection group (*P<0.01*). At day 9, there was no significant difference among these three groups in ADPN protein expression.

Effects of adiponectin on differentiation and lipid metabolism of cultured chicken preadipocytes

**Figure 2:**
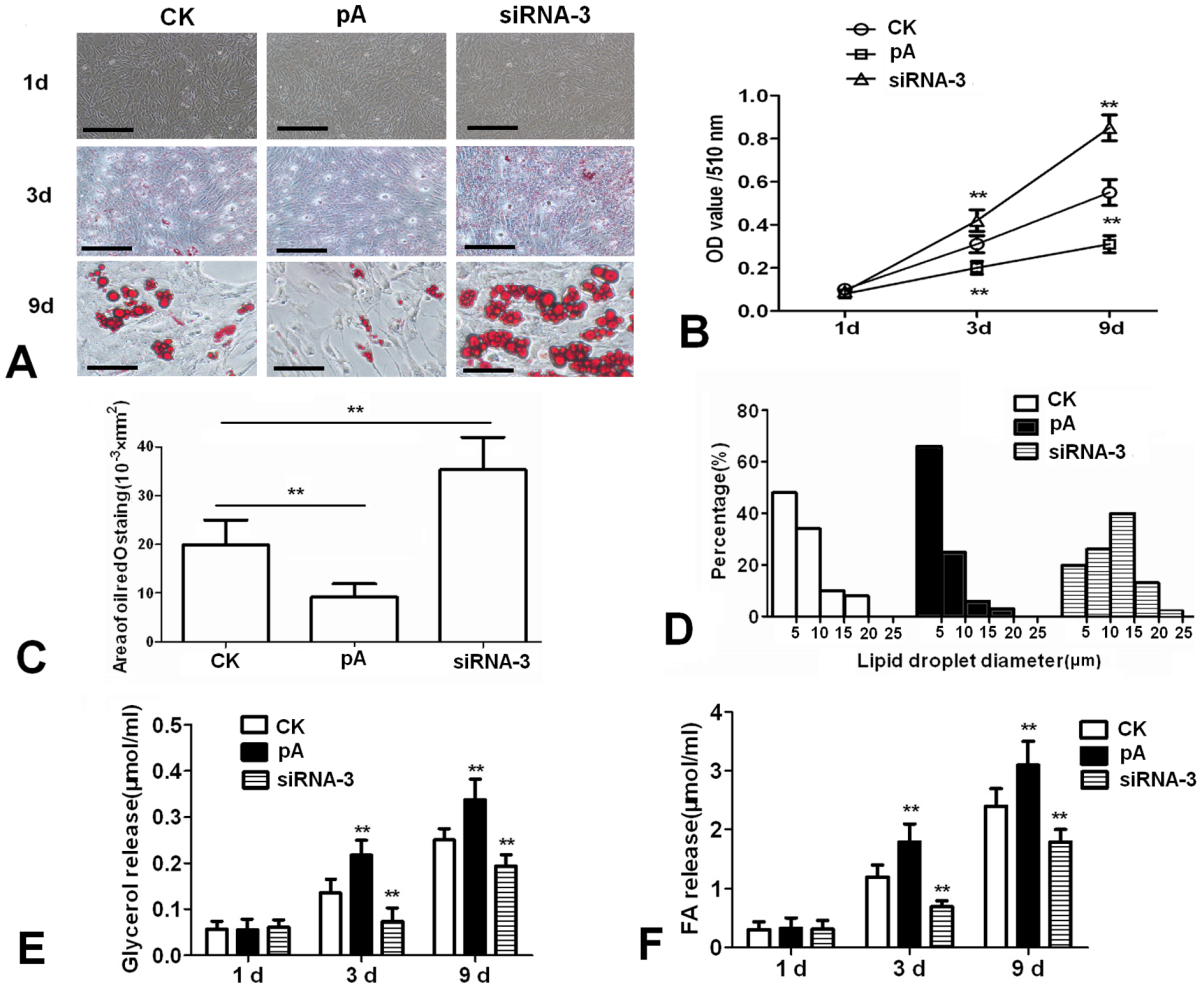
Effects of adiponectin on morphology changes and lipid metabolism of cultured chicken preadipocytes. (A) Representative images of Oil Red O-stained sections of three groups at day 1, 3 and 9. Scale bar, 100 µm. (B) Lipid accumulation was assessed by the quantification of A_510_ in destained Oil Red O with isopropyl alcohol (n = 3). (C) The areas stained with Oil Red O assessed (n = 3). (D) Lipid droplet diameter frequency distributions for the three groups. Numbers on the x-axis represent the bins for droplets of specific sizes. (E) Glycerol content in the medium (n = 3). (F) FFA content in the cell culture medium (n = 3). FFA: free fatty acid, CK: Control group, pA: pcDNA3.1-ADPN, siRNA-3: pGPU6/GFP/Neo-ADPN-952. Values are means ± SEM. vs. control group, * *P*<0.05, ** *P*<0.01.

The morphological changes of chicken adipocytes during cell culture periods were recorded. As shown in **Fig. 2A**, compared with control, Oil Red O staining showed that over-expression of adiponectin inhibited the lipid droplets formation at day 3 and 9, while siRNA-3 increased the lipid droplets formation. TG accumulation analysis in chicken adipocytes verified the results obtained on morphological changes, adiponectin decreased TG concentration in adipocytes at day 3 and 9 (*P*<0.01) (**Fig.** **2B**). The areas stained with Oil Red O analysis showed that over-expression of adiponectin reduced the total volume stained with Oil Red O (**Fig.** **2C**). Compared to the control group, pcDNA3.1-ADPN transfection group had more small droplets (<5 µm), whereas siRNA-3 increased the number of large droplets (>15 µm) (**Fig.** **2D**). In addition, glycerol and FFA content markedly increased in the cell culture medium in the pcDNA3.1-ADPN transfection group, while decreased in the siRNA-3 transfection group (*P*<0.01) (**Fig.** **2E & F**).

Effects of adiponectin on the expression levels of the lipid metabolism related genes

**Figure 3:**
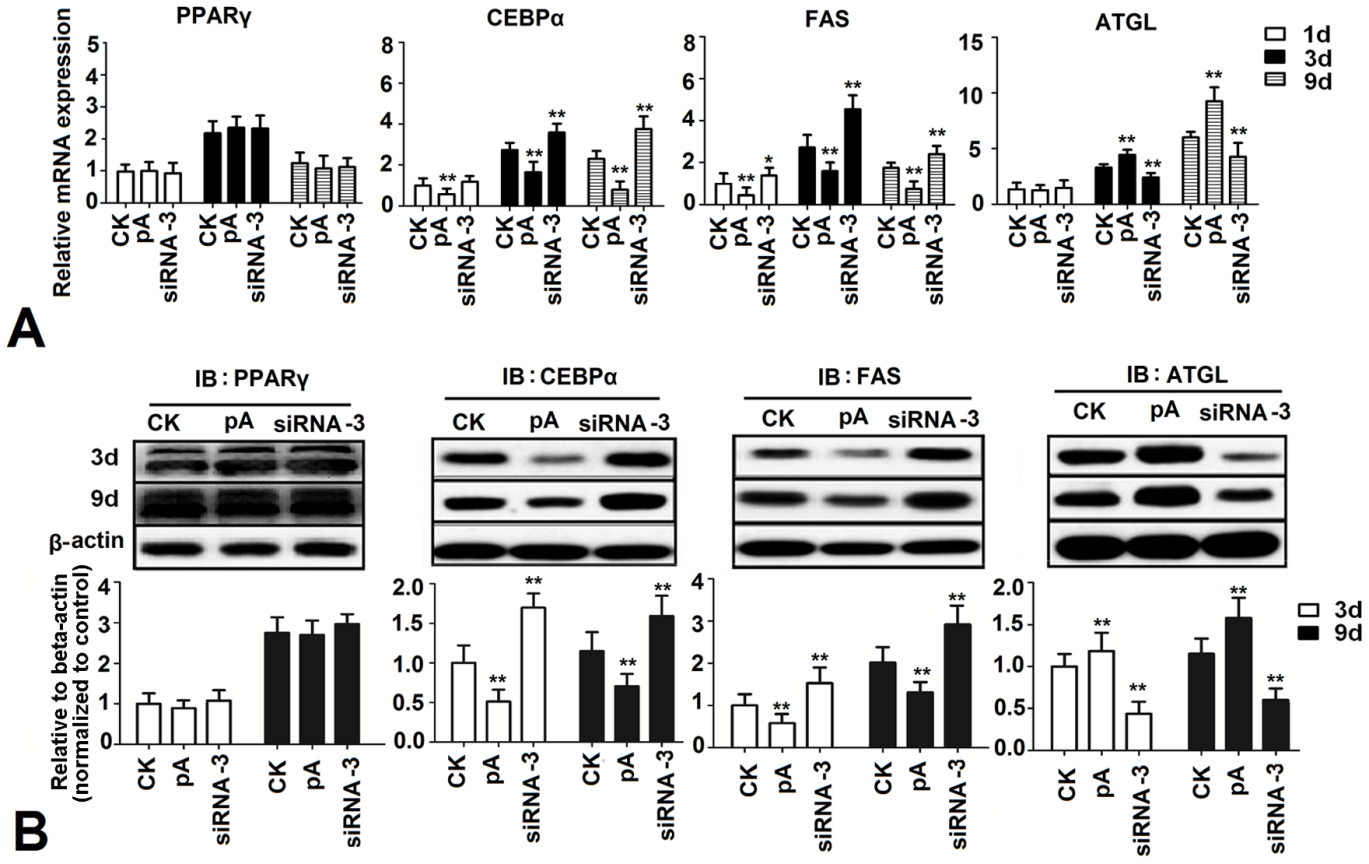
Effects of adiponectin on sequential expression of lipogenesis genes and proteins. (A) Expression levels of adipogenesis genes at day 1, 3 and 9 after transfection with pcDNA3.1-ADPN and siRNA-3 (n = 3). (B) Expression levels of adipogenesis proteins at day 1, 3 and 9 after transfection with pcDNA3.1-ADPN and siRNA-3 (n = 3). CK: Control group, pA: pcDNA3.1-ADPN, siRNA-3: pGPU6/GFP/Neo-ADPN-952. Values are means ± SEM. vs. control group, * *P*<0.05, ** *P*<0.01.

To examine the potential effects of adiponectin on lipogenesis in chicken adipocytes, real-time PCR was used to determine the expression levels of *PPARγ*, *C/EBPα*, *FAS* and *ATGL*. Data showed that when compared with control, over-expression of adiponectin significantly decreased the expression of *C/EBPα* and *FAS* at day 1,3 and 9, while increased the expression of *ATGL* at day 3 and 9 (*P*<0.01). siRNA-3 significantly increased the expression of *C/EBPα* and *FAS* at day 1,3 and 9, while decreased the expression of *ATGL* at day 3 and 9 (*P*<0.01). Adiponectin had no significant effect on the expression of *PPARγ* (*P*>0.05) (**Fig. 3A**). Results of western blot showed that, at day 3 and 9, over-expression of adiponectin significantly decreased the expression of C/EBPα and FAS, while increased the expression of ATGL (*P*<0.01). Moreover, siRNA-3 up-regulated the expression of C/EBPα and FAS, while down-regulated ATGL expression (*P*<0.01) (**Fig.** **3B**).

Over-expression of adiponectin activated p38 MAPK/ATF-2 pathway in chicken adipocytes

**Figure 4:**
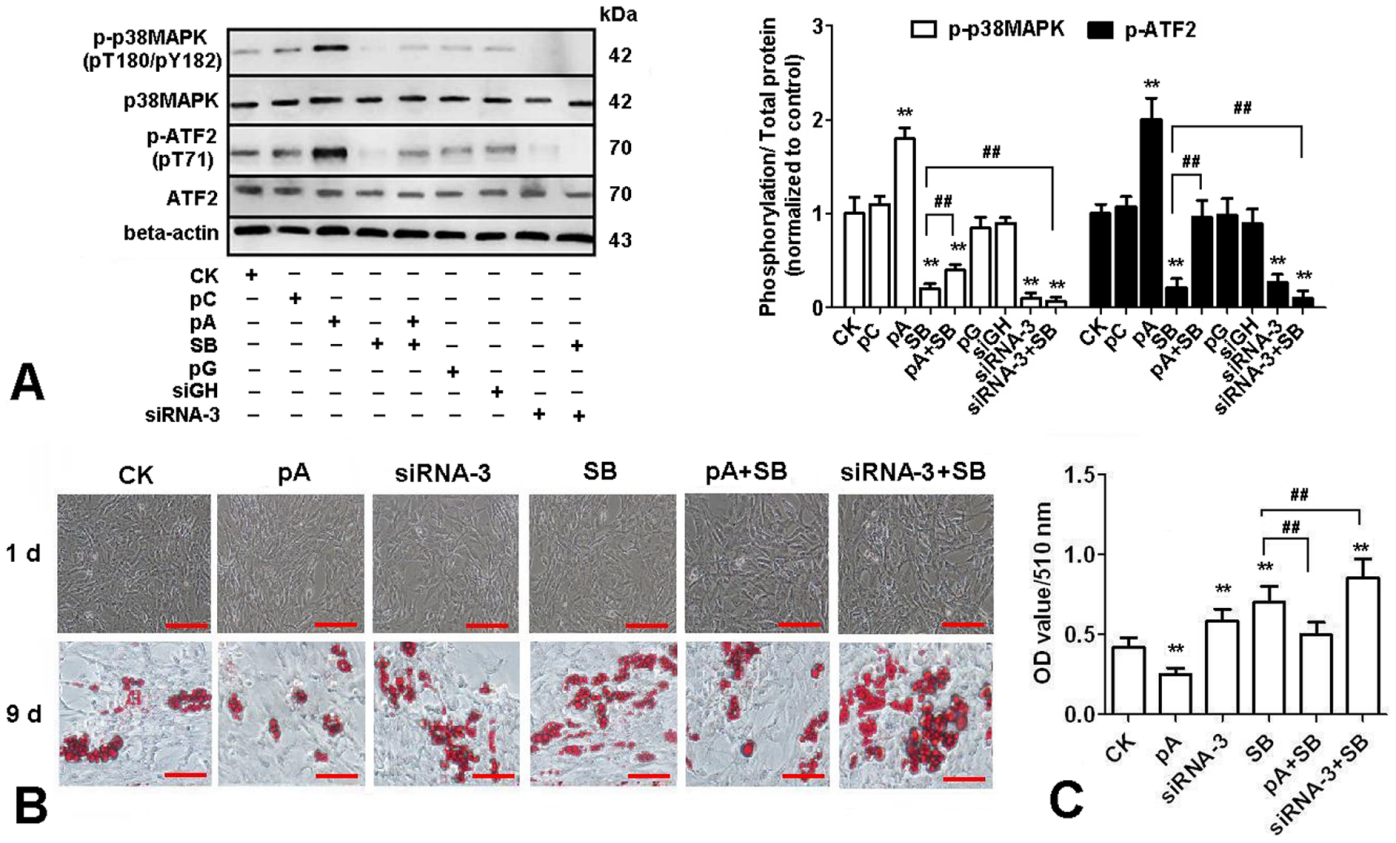
Adiponectin activates the p38 MAPK/ATF-2 pathway in cultured chicken preadipocytes. (A) Cells were treated either with recombination vectors alone or with 10 µM SB253580(SB), total proteins were extracted at 30 min after administration of SB253580 and then immunoblotted for total p38MAPK, phospho-p38MAPK (pT180/pY182), total ATF-2 and phospho-ATF-2 (pT71) (n = 3). (B) Representative images of Oil Red O-stained sections of cells at d 9 after treated either with recombination vectors alone or with 10 µM SB253580. (C) Lipid accumulation was assessed by the quantification of A_510_ in destained Oil Red O with isopropyl alcohol (n = 3). Scale bar, 100 µm. CK: Control group, pC: pcDNA3.1, pA: pcDNA3.1-ADPN, pG: pGPU6/GFP/Neo, siRNA-3: pGPU6/GFP/Neo-ADPN-952, siGH: pGPU6/GFP/Neo-GAPDH. Values are means ± SEM. vs. control group, * *P*<0.05, ** *P*<0.01. vs. SB253580 treatment group, # *P*<0.05, ## *P*<0.01.

To further characterize the underlying mechanisms for the effect of adiponectin on lipid metabolism, we used SB253580 (inhibitor of p38MAPK pathway) to treat chicken adipocytes after transfection with plasmids. As shown in **Fig.** **4A**, p38 MAPK and its downstream target-ATF-2 were activated as measured by phosphorylation with the over-expression of adiponectin, while the phosphorylation level decreased in siRNA-3 group (*P<0.01*). The morphology of chicken adipocytes at day 1 and 9 was recorded and TG concentration at day 9 was evaluated after Oil Red O staining with plasmids transfection. Morphological changes and TG concentration in adipocytes confirmed that p38 MAPK pathway mediated the lipid-lowering effects of the over-expression of adiponectin (**Fig.** **4B & C**). Data showed that at day 9, compared to the control group, over-expression of ADPN significantly inhibited lipid deposition in chicken adipocytes, while siRNA-3 and SB253580 significantly increased lipid deposition (*P*<0.01). Compared to SB253580 treatment group, lipid deposition decreased in the group co-treated with pCDNA3.1-ADPN and SB253580, while increased in the group co-treated with siRNA-3 and SB253580 (*P*<0.01).

Over-expression of adiponectin suppressed TOR/p70 S6 Kinase pathway in chicken adipocytes

**Figure 5:**
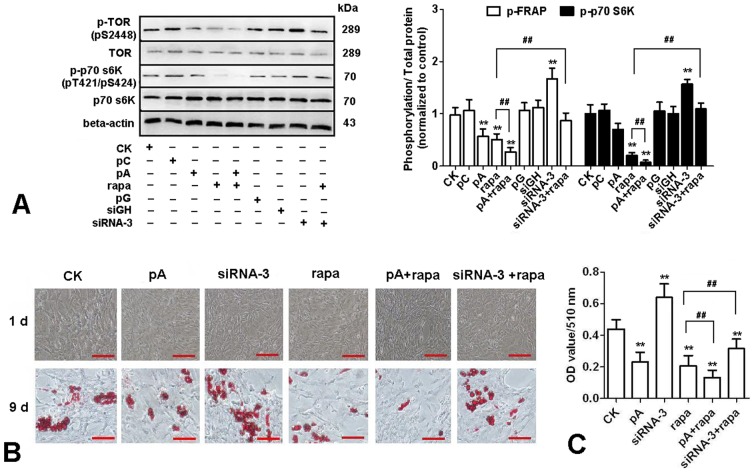
Adiponectin inhibited TOR/p70 S6 Kinase pathway in cultured chicken preadipocytes. (A) Cells were treated either with recombination vectors alone or with 10 nM rapamycin (rapa), total proteins were extracted 30 min after administration of rapamycin and then immunoblotted for total TOR, phospho-TOR (pS2448), total p70 S6Kinase and phospho-p70 S6Kinase (pT421/pS424) (n = 3). (B) Representative images of Oil Red O-stained sections of cells at d 9 after treated with recombination vectors alone or with 10 nM rapamycin. (C) Lipid accumulation was assessed by the quantification of A_510_ in destained Oil Red O with isopropyl alcohol (n = 3). Scale bar, 100 µm. CK: Control group, pC: pcDNA3.1, pA: pcDNA3.1-ADPN, pG: pGPU6/GFP/Neo, siRNA-3: pGPU6/GFP/Neo-ADPN-952, siGH: pGPU6/GFP/Neo-GAPDH. Values are means ± SEM. vs. control group, * *P*<0.05, ** *P*<0.01. vs. rapamycin treatment group, # *P*<0.05, ## *P*<0.01.

Rapamycin (inhibitor of TOR pathway) was also used to treat chicken adipocytes after transfection with plasmids. In **Fig.** **5A**, we found over-expression of adiponectin inhibited the activation of TOR and p70 S6Kinase, and pcDNA3.1-ADPN may further reduce the phosphorylation level of the TOR and p70 S6Kinase based on the inhibitory effect of TOR by rapamycin. Morphological changes and TG concentration in adipocytes confirmed that TOR/p70 S6 Kinase pathway mediated the lipid-lowering effects of adiponectin (**Fig.** **5B & C**). Results indicated that at day 9, compared to control group, over-expression of ADPN and rapamycin significantly inhibited lipid deposition in chicken adipocytes, while siRNA-3 significantly increased lipid deposition (*P*<0.01). Compared to rapamycin treatment group, lipid deposition decreased in the group co-treated with pCDNA3.1-ADPN and rapamycin, while increased in the group co-treated with siRNA-3 and rapamycin (*P*<0.01).

## Discussion

Adiponectin, an adipocytokine secreted by adipose tissue, has been confirmed to regulate glucose metabolism both *in vivo* and *in vitro* [3], [18]. There was a decrease in the blood level of adiponectin with excessive body fat deposition, while circulating adiponectin is raised with body fat loss, revealing that there is a close contact between adiponectin and body fat deposition[19], [20]. Tahmoorespur et al (2010) showed that adiponectin was inversely related to chicken belly fat deposition level [21]. The present study demonstrated that transfection with pcDNA3.1-ADPN reduced lipid deposition and increased lipolysis in chicken adipocytes. Conversely, the reduction in adiponectin secretion led to accelerated lipid accumulation. Masaki et al (2003) also reported that adiponectin reduced visceral adiposity in mice and they ascribed the mechanisms underlying reduction of body fat by adiponectin to increased energy expenditure [22]. These observations suggest that adiponectin functions as an adipocyte differentiation factor.

**Figure 6:**
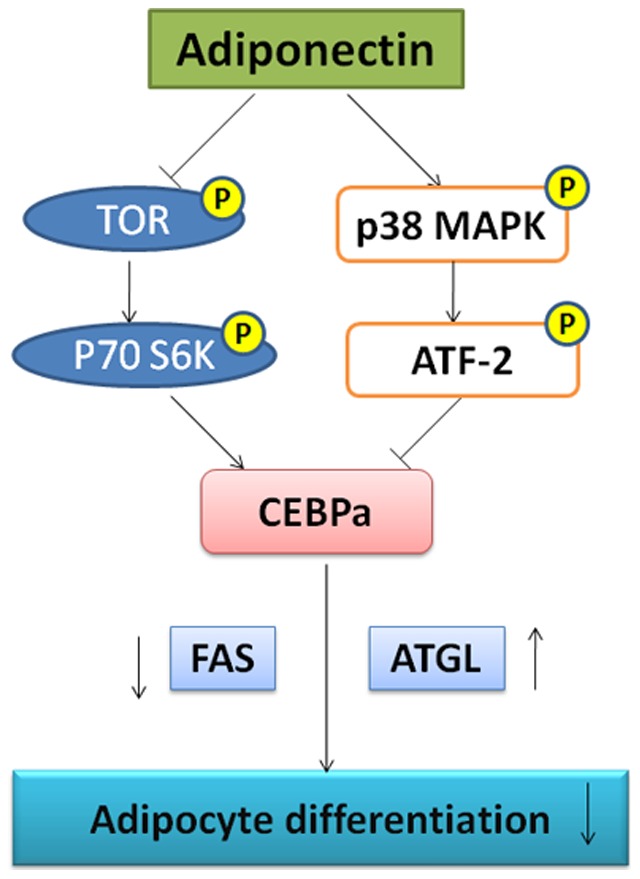
A summary of adiponectin's effect on anti-differentiation of chicken preadipocytes through p38 MAPK/ATF-2 and TOR/p70 S6 Kinase pathways. Adiponectin activates the p38 MAPK/ATF-2 pathway and inhibits the TOR/p70 S6 Kinase pathway, which reduce the expression of the transcription factor- CEBPα. With the reduction of CEBPα, the expression of FAS decreases and ATGL increases. Finally, lipid deposition and differentiation of chicken preadipocytes are inhibited.

The signaling pathways downstream of the adiponectin receptor are not yet well understood. Mao et al (2006) confirmed that AdipoR1/APPL1 mediated adiponectin signaling and its effects on metabolism and insulin sensitivity in liver, skeletal muscle and adipose. Adiponectin stimulates the interaction between APPL1 and Rab5 (a small GTPase), leading to increased GLUT4 membrane translocation. Consistent with their hypothesis, in cultured myotubes, APPL1 knockdown lowers adiponectin-stimulated fatty acid oxidation, glucose uptake, and phosphorylation of AMPK and p38 MAPK [23]. Herein we show that both p38 MAPK/ATF-2 and TOR/p70 S6 Kinase pathways contribute to the process of adiponectin induced chicken preadipocyte differentiation. Our findings are summarized in **Fig. 6**.

AMPK is a metabolic sensor that helps maintain cellular energy homeostasis [24]. Chabrolle et al (2007) reported that adiponectin increased phosphorylation of AMPK at Thr172 in a time-dependent manner in cultured chicken granulose cells [25]. Previously, AMPK also has been linked to inhibition of the TOR pathway [26], [27]. Yeh et al (1995) reported rapamycin, the TOR pathway inhibitor, inhibited the expression of C/EBPα and adipogenic differentiation of 3T3-L1 cells [28]. So we speculated that adiponectin mediated activation of AMPK may inhibit TOR in chicken preadipocytes. Phosphorylation of TOR and p70 S6 Kinase was immunoblotted to confirm the hypothesis. Results demonstrated that the phosphorylation of TOR and p70 S6 Kinase were decreased by over-expression of adiponectin. Rapamycin could further reduce the phosphorylation of TOR and p70 S6 Kinase, confirming that the physiological function of adiponectin reflected the activation status of TOR/p70 S6 Kinase.

Engelman et al (1998) showed that addition of the p38 inhibitors early in 3T3-L1 differentiation decreased adipocyte formation [29]. Activated p38 MAPK has been shown to phosphorylate the transcription factor ATF-2 [30]. Meanwhile, ATF-2 and C/EBPα can form a heterodimeric DNA binding complex *in vitro* and co-transfection of ATF-2 with C/EBPα results in decreased activation of transcription driven from consensus C/EBP-binding sites [31]. Recent studies suggested that p38 MAPK regulated adiponectin-induced glucose uptake and fatty acid oxidation in C2C12 myotubes and primary human hepatocytes [11], [32]. In this study, we certified that adiponectin suppressed chicken adipocyte differentiation through p38 MAPK/ATF-2 pathway.

In our research, over-expression of adiponectin reduced the area of Oil Red O stain and TG content in chicken adipocytes. Gene and protein expression analysis confirm the findings of fat accumulation *in vitro* and cell morphology. C/EBPα, a member of the basic region leucine zipper family of transcription factors, is involved in adipogenesis and hepatic glucose and lipid metabolism. It coordinately stimulates the expression of the adipocyte genes giving rise to the adipocyte phenotype. Previous studies reported that adiponectin is transcriptionally regulated by C/EBPα [33]. In this regard, we found that there was an inverse relationship between adiponectin and C/EBPα in chicken adipocyte. As a central enzyme in lipogenesis, the gene encoding fatty acid synthase was identified as a candidate gene for determining body fat [34]. In our study, the expression of FAS was negatively correlated with adiponectin *in vitro*. The decreased expression of FAS may play a role in the lower lipid accumulation in adipocytes. ATGL is an important factor involved in fat catabolism in adipose tissue [35]. The main function of ATGL is to catalyze triglyceride hydrolysis to glycerol diester [36]. In this study, the expression of ATGL was stimulated by adiponectin in cells, indicating that adiponectin could stimulate the function of lipolysis in chicken adipocyte.

In summary, our data showed that adiponectin inhibited lipid deposition and differentiation of chicken preadipocytes by suppressing the expression of CEBPα and FAS, while increasing the expression of ATGL. The mechanism is explained by the observation that adiponectin stimulates p38 MAPK and ATF-2 activation and suppresses the TOR/p70 S6 Kinase pathway.
